# A functional analysis of the pyrimidine catabolic pathway in Arabidopsis

**DOI:** 10.1111/j.1469-8137.2009.02843.x

**Published:** 2009-07

**Authors:** Rita Zrenner, Heike Riegler, Cathleen R Marquard, Peter R Lange, Claudia Geserick, Caren E Bartosz, Celine T Chen, Robert D Slocum

**Affiliations:** 1 Max Planck Institute of Molecular Plant Physiology14476 Potsdam OT Golm, Germany; 2 Leibniz-Institute of Vegetable and Ornamental Crops14979 Großbeeren, Germany; 3 Department of Biological Sciences, Goucher CollegeBaltimore, MD 21204-2794, USA

**Keywords:** *Arabidopsis*, catabolic pathway, mutant, nitrogen metabolism, pyrimidine nucleotide

## Abstract

- Reductive catabolism of pyrimidine nucleotides occurs via a three-step pathway in which uracil is degraded to β-alanine, CO_2_ and NH_3_ through sequential activities of dihydropyrimidine dehydrogenase (EC 1.3.1.2, PYD1), dihydropyrimidinase (EC 3.5.2.2, PYD2) and β-ureidopropionase (EC 3.5.1.6, PYD3).
- A proposed function of this pathway, in addition to the maintenance of pyrimidine homeostasis, is the recycling of pyrimidine nitrogen to general nitrogen metabolism. *PYD* expression and catabolism of [2-^14^C]-uracil are markedly elevated in response to nitrogen limitation in plants, which can utilize uracil as a nitrogen source.
- *PYD1*, *PYD2* and *PYD3* knockout mutants were used for functional analysis of this pathway in *Arabidopsis*. *pyd* mutants exhibited no obvious phenotype under optimal growing conditions. *pyd2* and *pyd3* mutants were unable to catabolize [2-^14^C]-uracil or to grow on uracil as the sole nitrogen source. By contrast, catabolism of uracil was reduced by only 40% in *pyd1* mutants, and *pyd1* seedlings grew nearly as well as wild-type seedlings with a uracil nitrogen source. These results confirm PYD1 function and suggest the possible existence of another, as yet unknown, activity for uracil degradation to dihydrouracil in this plant.
- The localization of PYD-green fluorescent protein fusions in the plastid (PYD1), secretory system (PYD2) and cytosol (PYD3) suggests potentially complex metabolic regulation.

## Introduction

Pyrimidine nucleotides are precursors in the synthesis of DNA and RNA, and pyrimidine nucleoside diphosphate sugars are activated intermediates in the synthesis of lipids, sucrose and cell wall polysaccharides (Zrenner *et al*., 2006). Pyrimidine nucleotides are synthesized from uridine-5′-monophosphate (UMP) via phosphotransfer and interconversion reactions. The salvaging of preformed nucleobases or nucleosides is also a major route for the synthesis of UMP and other pyrimidines (Wasternack, 1982; Moffatt & Ashihara, 2002; Stasolla *et al*., 2003).

**Fig. 1:**
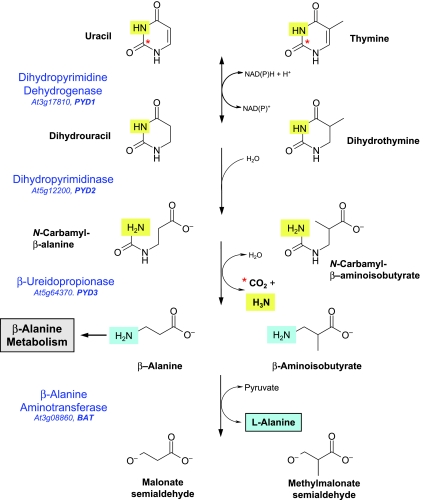
The reductive pathway for the degradation of pyrimidine nucleotides in Arabidopsis. Names of enzymes catalysing each reaction are given with the AGI locus and gene name. The asterisk indicates C-2 of the pyrimidine ring, which is released as CO_2_ in the PYD3 reaction. The N-3 pyrimidine nitrogen (light highlighting) is also released as ammonia at this step. The fourth step, in which the putative β-alanine aminotransferase (BAT) transfers pyrimidine N-1 (darker highlighting) to pyruvate or 2-oxoglutarate to form l-alanine or l-glutamate, respectively, is not considered to be part of the reductive pathway. It is included here to illustrate the route by which both pyrimidine nitrogen atoms are assimilated into general nitrogen metabolism.

In many bacteria, animals and plants (Wasternack, 1978; Rawls, 2006; Piskur *et al*., 2007), pyrimidine catabolism occurs via the ‘reductive’ pathway shown in Fig. 1. In this pathway, uracil (Ura) or thymine (Thy) degradation is initiated by dihydropyrimidine dehydrogenase (DHPDH, EC 1.3.1.2, PYD1) through NAD(P)H-dependent reduction of the C-5, C-6 double bond. The dihydrouracil (DHU) product of Ura reduction is hydrolytically degraded by dihydropyrimidinase (DHPase, EC 3.5.2.2, PYD2) to β-ureidopropionate (*N*-carbamyl-β-alanine), which is further metabolized to β-alanine, CO_2_ and NH_3_ by β-ureidopropionase (β-UPase, EC 3.5.1.6, PYD3). Thy catabolism to β-aminoisobutyrate (β-ΑΙΒ) in this pathway, with dihydrothymine and N-carbamyl-β-AIB intermediates, is also shown in Fig. 1. In Arabidopsis, each of the enzymes catalysing this three-step pathway is encoded by a single gene (Zrenner *et al*., 2006).

In plants and bacteria, β-alanine may be further metabolized to pantothenate (vitamin B_5_), an essential precursor in coenzyme A (CoA) synthesis (Coxon *et al*., 2005). β-Alanine is also required for the synthesis of β-alanine betaine, a compatible solute in some plants (Duhazé*et al*., 2003). β-Alanine and β-ΑΙΒ may further serve as amino donors, with 2-oxoglutarate or other amino acceptors, in β-alanine aminotransferase (BAT, EC 2.6.1.2)-catalysed reactions. In Arabidopsis, eight genes encode BAT, three of which have been functionally characterized (Liepman & Olsen, 2003). *In vitro*, plant BATs utilize a range of amino donors and acceptors, suggesting multiple, but largely unidentified, roles in metabolism (Liepman & Olsen, 2003). We have chosen At3g08860 as ‘*BAT*’ in the present study, as its expression is highly coordinated with the expression of the *PYD1*, *PYD2* and *PYD3* genes, and it is expressed at levels that are several-fold higher than for the other *BAT* genes. The entire pathway would serve to recycle both the N1 and N3 atoms of the pyrimidine ring to general nitrogen metabolism (Fig. 1).

The reductive pathway in higher plants was first demonstrated by Evans & Axelrod (1961) and Tsai & Axelrod (1965), who reported the presence of labelled DHU, β-ureidopropionate and β-alanine + ^14^CO_2_ products of [2-^14^C]-Ura catabolism in *Brassica napus* seedlings. Tsai & Axelrod (1965) also showed that Ura-derived β-alanine was used in both the synthesis of pantothenate and, via BAT activities utilizing a 2-oxoglutarate amino acceptor, the synthesis of l-glutamate + malic acid, confirming the extended catabolic pathway scheme (Fig. 1). Labelling kinetic studies have shown that the Ura → DHU (PYD1) step of the pathway is rate limiting for [2-^14^C]-Ura degradation in tomato (Tintemann *et al*., 1985), and thus may represent an important site of regulation. Although DHPDH activities have been inferred from the above studies, they have not been detected in any plant, and the possible role of this enzyme in pyrimidine catabolism has not been elucidated. By contrast, Arabidopsis cDNAs encoding DHPase and β-UPase have been shown to functionally complement *Saccharomyces kluyveri pyd2* and *pyd3* mutants (Gojkovic *et al*., 2001, 2003), and corn β-UPase was purified and characterized after heterologous expression in *Escherichia coli* (Walsh *et al*., 2001). Recently, a completely different and unrelated *PYD3* gene encoding β-alanine synthase from *S. kluyveri* has been described (Andersen *et al*., 2008a). The *Arabidopsis thaliana* genome encodes a relative to this gene [At5g43600, putative *N*-carbamyl-l-amino acid amidohydrolase, also annotated as *N*-acetylornithine deacetylase (ArgE) homologue (Slocum, 2005)], but its involvement in pyrimidine degradation in plants needs to be determined.

Numerous studies have confirmed a highly active catabolic pathway in plants. For example, Lesley *et al*. (1980) reported that both thymidine and Thy were rapidly (40 min) and extensively (50–60% total label in β-AIB + ^14^CO_2_) degraded, with little incorporation into DNA in sugarcane cells. In *Catharanthus* cells, 64% of [2-^14^C]-Ura and 94% of [2-^14^C]-Thy were catabolized (Sasamoto *et al*., 1987). In corn seedlings, [2-^14^C]-labelled Ura, DHU and Thy were all rapidly catabolized (Walsh *et al*., 2001). The relative importance of this pathway in maintaining pyrimidine homeostasis during plant growth and development, or in response to conditions that have an impact on nucleotide metabolism, such as phosphate limitation (Hewitt *et al*., 2005), is poorly understood. Its possible role in the recycling of pyrimidine nitrogen to general nitrogen metabolism has not been investigated in plants. Virtually nothing is known about the subcellular localization of catabolic pathway enzymes or the metabolic and genetic mechanisms regulating their activities.

In this study, a functional analysis of the catabolic pathway, employing T-DNA knockouts of the *PYD1*, *PYD2* and *PYD3* genes, was carried out. Expression profiling for *PYD* genes, and genes encoding other enzymes of pyrimidine metabolism and nucleobase transporters in both wild-type and *pyd* mutants, suggests that changes in pyrimidine metabolism and catabolic pathway activities are highly coordinated. We further demonstrate the subcellular localization of this pathway using PYD-green fluorescent protein (GFP) fusions, and provide evidence that recycling of pyrimidine nitrogen to general nitrogen metabolism is a major function of this pathway under conditions of nitrogen limitation.

## Materials and Methods

### Plant materials and growth conditions

*Arabidopsis thaliana*(L.) Heynh Columbia-0 (Col), SALK_083897 (*pyd1-2*), SALK_038919 (*pyd2-1*) and SALK_016594 (*pyd3*) were obtained from the Nottingham Arabidopsis Stock Centre (University of Nottingham, Loughborough, UK), and GABI_251F09 (*pyd1-1*) and GABI_114F11 (*pyd2-2*) from the GABI-Kat collection (Max-Planck-Institut für Züchtungsforschung, Cologne, Germany). Seeds were surface sterilized and grown aseptically on 1/2 × Murashige and Skoog (MS) medium including vitamins (Murashige and Skoog, 1962), 0.25 mm 2-[*N*-morpholino]ethanesulphonic acid (MES), pH 5.7 (KOH), 0.5% (w/v) sucrose and 0.7% (w/v) agar, or in the same liquid medium (no agar) in shaking cultures (100 rpm). Seeds were imbibed at 4°C in the dark for 48 h and grown in a 12-h photoperiod (photon flux density, 150 µmol m^−2^ s^−1^; 22°C light; 18°C dark).

For some experiments with Ura as nitrogen source, plants were grown for 38 d aseptically under the same conditions as above, either without nitrogen or with 1 mm Ura as the sole nitrogen source. In other experiments, plants were grown for 6 wk on washed vermiculite potting medium and fertilized once a week with equal volumes of the above plus 3 mm nitrogen, 0.4 mm nitrogen, no nitrogen or 1 mm Ura liquid medium, and otherwise watered with distilled H_2_O. Plants were grown in a 16-h photoperiod (photon flux density, 120 µmol m^−2^ s^−1^; 60% relative humidity; 20°C light; 18°C dark).

### Bioinformatic analysis

Sequences were obtained from The Arabidopsis Information Resource (TAIR) (http://www.arabidopsis.org) and National Center for Biotechnology Information (NCBI) (http://www.ncbi.nlm.nih.gov). Sequence comparisons were performed with ClustalW software (http://www.embl.co.uk). For predicting the intracellular localization, the software packages Target P v.1.1 (http://www.cbs.dtu.dk/services/TargetP/; Emanuelsson *et al*., 2007), iPSORT (biocaml.org/ipsort/iPSORT/; Bannai *et al*., 2002) and Predotar v.1.03 (urgi.versailles.inra.fr/predotar/predotar.html; Small *et al*., 2004) were used.

### RNA isolation and expression analyses

For semi-quantitative reverse transcriptase-polymerase chain reaction (RT-PCR), total RNA was isolated from liquid-cultured seedlings or rosette leaves of vermiculite-grown seedlings using an RNeasy Kit (Qiagen, Valencia, CA, USA) followed by DNaseI treatment (DNA-free, Ambion, Austin, TX, USA). Gene-specific primer sets are listed in Table S1 (see Supporting Information). Relative transcript levels were normalized on the basis of the expression of the *ACT2* invariant control and quantified by densitometric analyses using ImageJ (Abramoff *et al*., 2004). For quantitative real-time RT-PCR, total RNA was isolated from 16-d-old sterile culture-grown seedlings using a NucleoSpin Plant Kit (Macherey-Nagel GmbH and Co KG, Düren, Germany), including on-column DNaseI digestion. Single-stranded cDNA synthesis was carried out with total RNA using SuperScript™ III RNaseH^−^ reverse transcriptase (Invitrogen GmbH, Karlsruhe, Germany). Quantitative two-step RT-PCR was performed using a SYBR® Green 1 protocol (Wittwer *et al*., 1997) and a 7900HT Fast Real-Time PCR System (Applied Biosystems, Foster City, CA, USA). The gene-specific primer pairs used in these analyses are listed in Table S2 (see Supporting Information).

### Construction of PYD-GFP fusion proteins

Entire *PYD1*, *PYD2* and *PYD3* open reading frames (ORFs) were amplified from first-strand cDNA by PCR with *Pfu*-polymerase (MBI Fermentas, St Leon-Roth, Germany) using the following oligonucleotides: Pyd1_5, CACCATGGCTTCCATGAGTTTCGCC; Pyd1_3, GTTAGAAACCATACTCTCAGTCT; Pyd2_5, CACCATGGCTCTGGATGCATTCTTCT; Pyd2_3, CGTAGCTTCAGTTCTGACACG; Pyd3_5, CACCATGGATCATATGATATCAGAAAAC; Pyd3_3, TGTAGAATTCTTGTGGAGCAATG; PCR products were inserted into entry vector pENTR™/SD/D-TOPO® (Invitrogen GmbH) and positive entry clones were confirmed by sequencing (MWG Biotech AG, Ebersberg, Germany). Clones were recombined into pK7FWG2 plant transformation vector for C-terminal GFP fusion (Karimi *et al*., 2002). Constructs were transferred into Arabidopsis by *Agrobacterium*-mediated transformation using the floral dip method (Clough & Bent, 1998). Transgenic plants were selected by kanamycin resistance. Confocal microscopy was performed on 4–6-wk-old plants grown on soil. Stably transformed Arabidopsis plants expressing AMK2-GFP (Lange *et al*., 2008) were used as plastid control, those expressing *mgfp4*-ER (Haseloff, 1998) were used as endoplasmic reticulum (ER) control, and those expressing GFP alone were used as cytosolic control.

### Isolation of *pyd* mutants

Plants with insertions in *PYD1* (*pyd1-1*, GABI_251F09; *pyd1-2*, SALK_083897), *PYD2* (*pyd2-1*, SALK_038919; *pyd2-2*, GABI_114F11) and *PYD3*(*pyd3*, SALK_016594) were obtained from the Salk collection (Alonso *et al*., 2003) and the GABI-Kat services (Rosso *et al*., 2003). Screening and selection within the mutant population were performed following the Signal Salk/GABI-Kat instructions (http://www.signalsalk.edu and http://www.mpiz-koeln.mpg.de/GABI-Kat/General_Information). Genomic DNA was isolated using an alkaline lysis protocol (Klimyuk *et al*., 1993) from a small leaf removed from third- or fourth-generation plants, and PCR genotyping was performed using T-DNA LB (left border)-specific primers and the gene-specific primer pairs shown in Table S3 (see Supporting Information). Homozygous mutants for each of the lines were isolated from selfed populations of the respective mutant.

### Construction and isolation of *PYD1* RNA interference (RNAi) lines

A 306-bp *PYD1* sequence was amplified by PCR from DNaseI-treated, reverse-transcribed total RNA using the primers 5′-CGGGATCCATCTCTCTAATGG-3′ (*Bam*HI site) and 5′-CTAGCTAGCATCTAAAGATACGG-3′ (*Nhe*I site) that spanned the portion of the ORF corresponding to the N-terminal amino acid residues 1–98. The *Bam*HI/*Nde*I restriction fragment was cloned into the *Bam*HI and compatible *Xba*I sites of the shuttle vector pRNA69 in the sense (S) orientation. The same sequence was amplified using primers 5′-ACGCGTCGACATCTAAAGATACGG-3′ (*Sal*I site) and 5′-CGGAATTCATCTCTCTAATTATGG-3′ (*Eco*RI site), and the *Sal*I/*Eco*RI restriction fragment was cloned into the compatible *Xho*I and *Eco*RI sites of pRNA69 in the anti-sense (AS) orientation. A *Not*I-flanked expression cartridge (35S promoter:AS insert:YABBY5 intron:S insert:ocs-3′ termination sequence) was cloned into the *Not*I site of the binary vector pMLBart, followed by *Agrobacterium*-mediated transformation of Arabidopsis (Col-0) seedlings, selection of Basta-resistant plants, and PCR verification of T-DNA inserts containing the RNAi expression cartridge in genomic DNA, using sets of gene-specific and vector-specific primers. The details of these procedures have been described by Chen & Slocum (2008). RT-PCR using the *PYD1* primers in Table S1 was used to estimate relative PYD1 silencing in RNAi lines.

### [2-^14^C]-Ura metabolism

Studies monitoring Ura metabolism in seedlings were carried out with liquid cultures as follows. One hundred *Arabidopsis* seeds were surface sterilized and imbibed at 4°C for 2 d in the dark. Seeds were transferred into 30 ml of sterile liquid culture medium in 250-ml Erlenmeyer flasks on orbital shakers with constant fluorescent light (photon flux density of 50 µmol m^−2^ s^−1^ in the flask) at 22°C. The medium contained 2 mm KNO_3_, 1 mm NH_4_NO_3_, 1 mm glutamine, 3 mm KH_2_PO_4_/K_2_HPO_4_ at pH 5.8, 4 mm CaCl_2_, 1 mm MgSO_4_, 2 mm K_2_SO_4_, 3 mm MES at pH 5.8 (KOH), 0.5% (w/v) sucrose, 40 µm Na_2_FeEDTA, 60 µm H_3_BO_3_, 14 µm MnSO_4_, 1 µm ZnSO_4_, 0.6 µm CuSO_4_, 0.4 µm NiCl_2_, 0.3 µm HMoO_4_ and 20 nm CoCl_2_. A low shaker speed during the first 2 d (30 rpm) was increased to 80 rpm. At least three replicates were made for each genotype. After 7 d of growth, seedlings were fed with Ura for 18 h under the same growth conditions by adding 1 µCi [2-^14^C]-Ura (7.4 GBq mmol^−1^; Hartmann Analytic GmbH, Braunschweig, Germany) and unlabelled Ura to a final concentration of 2 mm in each flask. In order to capture ^14^CO_2_ that is released during [2-^14^C]-Ura degradation, filter discs of Whatman 3MM paper were soaked with 4 m KOH and placed on top of each flask. Seedlings were harvested and washed three times with incubation medium containing 5 mm unlabelled Ura. After fresh weight determination, seedlings were frozen in liquid nitrogen. The radioactivity of the filter papers was eluted for 12 h in 10 ml of water. Frozen plant material was powdered using a ball mill (Retsch, Haan, Germany). Radiolabelled metabolites were extracted from 250 mg of frozen plant material by homogenization in 750 µl of cold 10% (v/v) perchloric acid (PCA) containing 10 mm ethylene glycol tetraacetic acid (EGTA), and allowed to incubate for 30 min on ice. After centrifugation at 16 000 ***g*** for 5 min at 4°C, the supernatant was removed and saved. The pellet was washed twice with 250 µl of cold 80% (v/v) ethanol, and then all three supernatants were combined and neutralized using 5 m KOH containing 1 m triethanolamine. The washed pellet was counted directly.

In other experiments, labelled Ura metabolism was assayed using rosette leaf tissue harvested from 6-wk-old plants, as follows. Tissue was placed in 1 ml of 10 mm potassium phosphate buffer, pH 5.8 containing 1 mm unlabelled Ura + 1 µCi [2-^14^C]-Ura (7.4 GBq mmol^−1^; Sigma-Aldrich Co., St. Louis, MO, USA) within capped 75 × 12-mm polypropylene tubes. A parallel set of samples was set up, in which a 2 m KOH-saturated, 6 mm Whatman 3MM paper disc was mounted inside the tube on a plugged 22-gauge syringe needle, which was inserted through the tube cap. Both sets of samples were processed, as described above, with the exception that the ^14^CO_2_ capture assay was terminated by the addition of 1 ml of 10% (v/v) PCA through the syringe, followed by recapping of the tube and the capture of released ^14^CO_2_ for an additional 6 h. PCA-insoluble pellet materials were also hydrolysed overnight in 6 m HCl before neutralization and counting of radioactivity in the 16 000 ***g*** supernatant. Radioactivity from incorporated [2-^14^C]-Ura in the catabolic (^14^CO_2_) fraction, soluble fraction [unmetabolized Ura, catabolic pathway intermediates, nucleobases, nucleotides, uridine diphosphate (UDP)-sugars] and insoluble fraction (DNA and RNA), as well as the unincorporated label remaining in the medium and seedling washes, was determined using an LS6500 Multi-Purpose Scintillation Counter and Ready Safe scintillation cocktail (Beckman-Coulter, Krefeld, Germany).

### Analyses of nucleotides, nucleosides and nucleobases

Plant material was quickly frozen in liquid nitrogen, extracts were made and nucleotides were measured as in Schröder *et al*. (2005). Nucleosides and nucleobases were separated using reversed-phase high-performance liquid chromatography (HPLC) (Adsorbosphere Nucleotide-Nucleoside 7 µm column, 250 × 4.6 mm; Alltech Associates, Deerfield, IL, USA) and a multistep gradient with solvent A (20 mm KH_2_PO_4_, 5 mm tetrabutylammonium phosphate) and B (100% methanol) as follows: 11 min 5% B, 19 min 13% B, 12 min 44% B, 8 min 5% B (Shannon *et al*., 1996). Nucleobases and nucleosides were identified by co-chromatography with authentic standards using a photodiode array detection method (absorption at 254 nm for quantification). As uridine in plant extracts did not elute as a pure peak, it was additionally identified and quantified after conversion to Ura. Therefore, the *UDP* gene encoding uridine phosphorylase of *E. coli* (GeneID: 948987) was cloned and over-expressed as a histidine (His)-tagged fusion protein in *E. coli*. Purification was performed using standard protocols for native His-tagged protein purification. Plant extracts were measured before and after incubation with the recombinant UDP protein. Unpaired two-tailed *t*-tests were used to compare pooled data from the different types of transgenic material.

## Results and Discussion

### Genes encoding enzymes for *de novo* and salvage synthesis, catabolism and nucleobase transporters are coordinately regulated

**Fig. 2:**
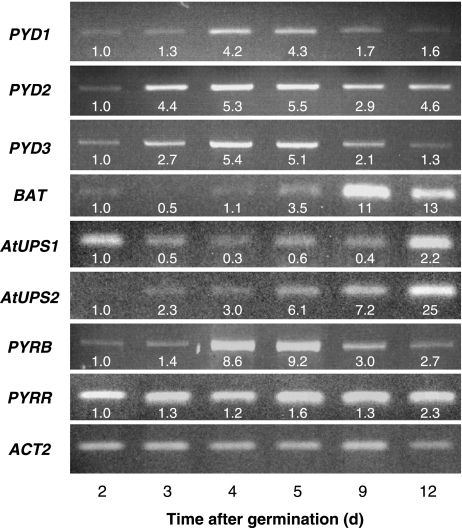
Reverse transcriptase-polymerase chain reaction (RT-PCR) expression profiles for genes encoding catabolic pathway enzymes (*PYD1–3, BAT*), nucleobase transporters with high affinity for uracil (*AtUPS1, AtUPS2*), the *de novo* synthesis pathway enzyme aspartate transcarbamoylase (*PYRB*) and salvage enzyme uracil phosphoribosyltransferase (*PYRR*). Expression values for each transcript, relative to Day 2 and normalized on the basis of constitutively expressed *ACT2*, are indicated below each band. Arabidopsis seedlings were grown in liquid culture and harvested at the indicated number of days after germination.

During the first few days after germination, metabolic demands for seedling growth appear to be met primarily by the salvaging of nucleobases from seed reserves, followed by increased *de novo* synthesis (Stasolla *et al*., 2003). As shown in Fig. 2, expression of *PYRR*, encoding the salvage enzyme uracil phosphoribosyl transferase (UPRTase), is relatively high and remains unchanged between Days 2 and 9, increasing slightly by Day 12. Nucleobase transporters AtUPS1 and AtUPS2 are localized in vascular tissues and function as the major Ura transporters in this plant (Schmidt *et al*., 2004). High expression of the Ura transporter *AtUPS1* during the first day after germination may facilitate initial nucleobase salvaging from seedlings (Schmidt *et al*., 2004), although its expression subsequently decreases *c*. twofold until Day 12, when transcript levels increase *c*. fourfold. By contrast, the expression of *AtUPS2* is barely detectable at germination, but increases dramatically over the following week, with Day 12 transcript levels being 25-fold higher than on Day 2. The induction of *AtUPS2* expression in the first days after germination coincides with increased expression of *PYRB*, encoding the *de novo* synthesis enzyme aspartate transcarbamoylase, consistent with a role for the transporter in facilitating the movement of newly synthesized nucleobases throughout the plant in order to meet metabolic demands during growth. *PYRB* transcript levels increase *c*. ninefold by Days 4–5, and then decrease slowly, perhaps in response to the oversupply of nucleobases through combined salvaging activities and increased *de novo* synthesis. It is interesting to note that the expression profiles for the catabolic pathway genes *PYD1–3* and *BAT* parallel that of *PYRB*. On Day 2, the expression of *PYD1–3* and *BAT* is barely detectable. By Days 4–5, *PYD1–3* expression has increased by *c*. four- to fivefold, coinciding with peak *PYRB* expression. Between Days 5 and 12, *PYD2* expression remains constant, whereas *PYD1* and *PYD3* decrease *c*. threefold. An analysis of AtGenExpress data in the expression atlas of Arabidopsis development (Schmid *et al*., 2005) confirms that the expression of *PYD1* and *PYD3* remains coordinately regulated throughout plant development. Markedly increased *BAT* expression is observed between Days 9 and 12 in Arabidopsis seedlings (Fig. 2), as *PYD1* and *PYD3* expression begins to decrease, perhaps indicating that *BAT* may be regulated in response to intermediates or products of the catabolic pathway.

Coordinate regulation of*PYD1–3* with *BAT*supports the previous assumption that At3g08860 may be the BAT involved in pyrimidine catabolism. Coordinate regulation of*PYD1–3* and *BAT*with *PYRB* suggests that PYD activities increase in response to increasing pyrimidine levels in the tissue, resulting from increased *de novo* synthesis activities. Thus, a major function for the catabolic pathway may be to regulate cellular levels of free pyrimidine nucleotides during growth and development.

In addition to maintaining pyrimidine homeostasis, or recycling of nitrogen from pyrimidines (see below), it has been proposed that pyrimidine catabolism plays a major role in the production of β-alanine, the precursor for pantothenate and CoA/acyl carrier protein synthesis (Coxon *et al*., 2005). In animals, the catabolic pathway is the sole route for β-alanine synthesis (Traut & Jones, 1996), but, in plants, β-alanine production from polyamine oxidation (Terano & Suzuki, 1978) or other processes may be more important. Recently, Katahira & Ashihara (2006) have reported that [U-^14^C]-Ura is extensively degraded to ^14^CO_2_ in potato leaf tissues (37% total incorporated label). Although 8% of the label resided in β-alanine, only 1% of the radioactivity occurred in pantothenate and 21% of the label was found in common amino acids, presumably derived from labelled C-skeletons produced by BAT reaction (see Fig. 1). Similarly, Tsai & Axelrod (1965) reported that 72% and 88% of label from [1-^14^C]- and [2-^14^C]-β-alanine, respectively, were found in the lipid fraction in rape seedlings, being derived from the BAT reaction product malic acid, with further metabolism via an acetate intermediate. These findings suggest that β-alanine derived from Ura catabolism contributes little to pantothenate synthesis in plants, the vast majority of which is metabolized via BAT. The marked increase in *BAT*expression following increased*PYD1–3* expression in developing Arabidopsis seedlings (Fig. 2) is consistent with this model, and suggests that the primary function of the catabolic pathway is the recycling of nitrogen from pyrimidines to general nitrogen metabolism.

### The catabolic pathway efficiently recycles pyrimidine nitrogen to general nitrogen metabolism and is regulated in response to changes in nitrogen availability

The role of the catabolic pathway in recycling of pyrimidine nitrogen to general nitrogen metabolism was investigated by growing seedlings with or without nitrogen and with Ura as a sole nitrogen source, and examining the effects on catabolic pathway activities and *PYD1–3*and*BAT* expression. Seedlings grown in vermiculite and fertilized weekly with 1/2 × MS medium (3 mm total nitrogen) grew and developed normally, and flowered and set seed over a 6-wk period (Fig. S1A, see Supporting Information). Seedlings grown in medium containing only 0.4 mm nitrogen were much smaller and began to flower *c*. 1-wk earlier than the full nitrogen control plants (Fig. S1B). Seedlings fertilized with medium containing no nitrogen grew for *c*. 10 d, and then arrested as small rosette plants with little root growth. These plants did not flower and exhibited slight chlorosis (Fig. S1C). Fertilization of seedlings with the same solution, but containing 1 mm Ura as the sole nitrogen source, resulted in the growth of small plants with single flower bolts with only one or two siliques (Fig. S1D); however, completion of their development suggests that they were able to catabolize Ura relatively efficiently.

**Fig. 3:**
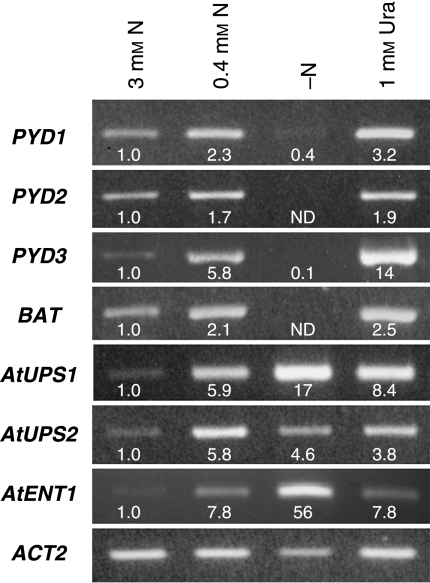
Reverse transcriptase-polymerase chain reaction (RT-PCR) analysis of gene expression in rosette leaf tissue of 6-wk-old Arabidopsis seedlings grown in vermiculite potting medium and fertilized with 1/2 × Murashige and Skoog (MS) medium containing 3 mm nitrogen, 0.4 mm nitrogen, no nitrogen (−N) or 1 mm uracil (Ura) as sole nitrogen source. *PYD1–3*, catabolic pathway; *BAT*, β-alanine aminotransferase; *AtUPS1* and *AtUPS2*, uracil transporters; *AtENT1*, uridine transporter; *ACT2*, constitutively expressed reference gene. Expression values for each transcript, relative to seedlings grown in 3 mm nitrogen and normalized on the basis of *ACT2*expression, are indicated below each band. ND, not detectable.

Gene expression profiles of rosette leaves for 6-wk-old seedlings in this experiment (Fig. 3) show that moderate nitrogen limitation in 0.4 mm nitrogen plants increased the expression of all four *PYD* genes between two- and sixfold, with the expression of the Ura transporters *AtUPS1* and *AtUPS2* also strongly up-regulated by *c*. sixfold. As shown in Table 1, 0.4 mm nitrogen plants incorporated nearly twice as much label from [2-^14^C]-Ura as plants grown in 3 mm nitrogen, consistent with the increased expression of nucleobase transporters. In 0.4 mm nitrogen plants, Ura catabolism also increased twofold, in parallel with increased expression of the *PYD* genes, although the distribution of label in catabolic, soluble and insoluble pools, expressed as a percentage of total incorporated Ura, was not different in 0.4 mm versus 3 mm nitrogen plants. Severe nitrogen limitation resulted in barely detectable *PYD1* and *PYD3* expression, and expression of *PYD2*and *BAT* was not evident (Fig. 3). In these plants, expression of *AtUPS1* and the nucleoside transporter *AtENT1* (Li *et al*., 2003) was even more strongly up-regulated than in 0.4 mm nitrogen plants (Fig. 3), although the total incorporated Ura and percentage Ura catabolism did not differ between these treatments (Table 1). By contrast, plants grown with 1 mm Ura incorporated 2.5-fold more labelled Ura than did plants grown in 3 mm nitrogen, and *c*. 20% more labelled Ura than plants grown without nitrogen (Table 1), despite a twofold decrease in *AtUPS1* and an approximate sevenfold decrease in *AtENT1* expression (Fig. 3). On a percentage total incorporated Ura basis, the 1 mm Ura plants catabolized nearly twice as much Ura as in other treatments (Table 1). Decreased label in soluble pools and increased label in CO_2_ and insoluble pools suggest that increased catabolism of Ura, with recycling of pyrimidine nitrogen to general nitrogen metabolism, in addition to efficient Ura salvaging into pyrimidines and nucleic acids, supported growth on Ura as a sole nitrogen source. The observed increase in catabolic activities in plants grown in 1 mm Ura was correlated with elevated *PYD* expression, with *PYD1* and *PYD3* transcript levels *c*. threefold and 14-fold higher than in 3 mm nitrogen plants and *c*. twofold higher than in 0.4 mm nitrogen plants (Fig. 3).

**Table 1:** [2-^14^C]-Uracil (Ura) metabolism in rosette leaves of Arabidopsis seedlings grown for 6 wk in vermiculite and watered weekly with equal volumes of 1/2 × Murashige and Skoog (MS) medium containing 3 mm total nitrogen (1 mm KNO_3_ and 1 mm NH_4_NO_3_; 3 mm nitrogen), 0.4 mm total nitrogen (0.13 mm KNO_3_ and 0.13 mm NH_4_NO_3_), no nitrogen (− N) or 1 mm Ura as the sole nitrogen source

	Uracil metabolism (nmol g^−1^ FW)			
Sample	Soluble	Insoluble	Catabolic (^14^CO_2_)	Total incorporated
3 mm N	603 ± 57 (75)	116 ± 9 (15)	77 ± 8 (10)	796.2 ± 73.5
0.4 mm N	1058 ± 85* (76)	207 ± 8*(15)	130 ± 8* (9)	1395 ± 100*
–N	1168 ± 92* (82)	145 ± 18 (10)	121 ± 10* (8)	1434 ± 119*
1 mm Ura	1180 ± 41* (60)	466 ± 108* (24)	325 ± 26* (16)	1970 ± 173*

Values are means ± standard error for four replicates. Values in parentheses indicate the percentage of total incorporated uracil. Soluble, perchloric acid-soluble fraction; Insoluble, perchloric acid-insoluble fraction.

* Significant differences (*P* < 0.05) from 3 mm nitrogen metabolite profiles using unpaired two-tailed *t*-tests.

A recent genome-wide expression analysis of primary and secondary metabolism in Arabidopsis seedlings confirmed that rapid changes in *PYD* expression also occur in response to changes in nitrogen availability (Scheible *et al*., 2004). In liquid-cultured seedlings, short-term, moderate nitrogen limitation leads to increased expression of all *PYD*genes and can be rapidly reversed (30 min to 3 h) by the addition of nitrate to the growth medium. Thus, it appears that both rapid and longer term adaptive changes in *PYD* expression occur in response to changes in nitrogen availability, and may play an important role in balancing the needs of pyrimidine and general nitrogen metabolism.

In senescing leaf tissues, *PYD1–3*and*BAT* are co-expressed at very high levels (AtGenExpress data on Arabidopsis development; Schmid *et al*., 2005) and catabolic pathway activities remain high even as salvaging activities decline in tobacco leaves (Ashihara, 1981). It seems likely that the release of pyrimidine nitrogen via the catabolic pathway would play a significant role in remobilization of nitrogen in senescent tissues (Hörtensteiner & Feller, 2002), but little appears to be known about this process.

### Regulation of *PYD* expression by catabolic pathway intermediates and products

**Fig. 4:**
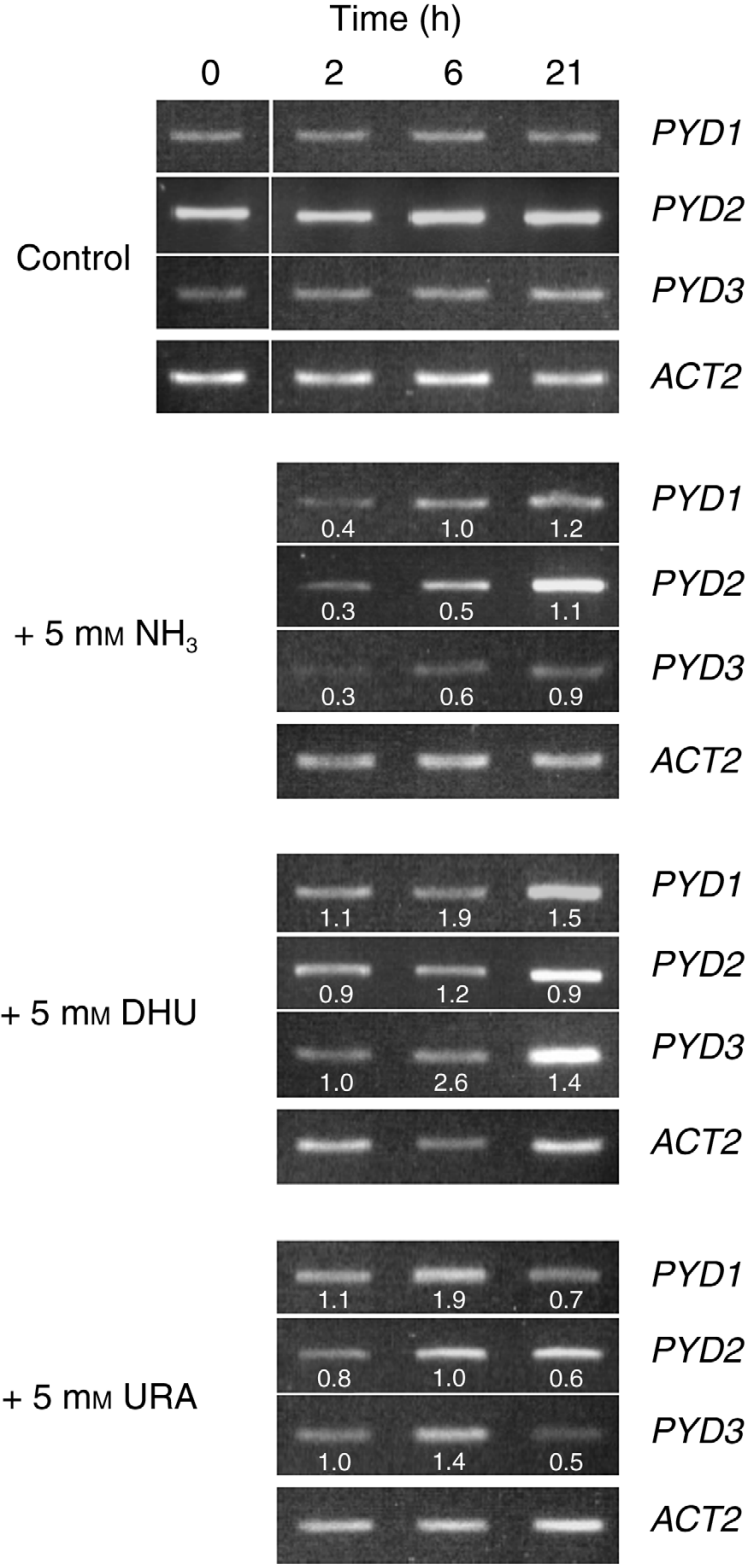
Reverse transcriptase-polymerase chain reaction (RT-PCR) expression profiles for *PYD1–3* in 17-d-old liquid-cultured Arabidopsis seedlings grown in 1/2 × Murashige and Skoog (MS) medium, with a fresh medium change at Day 9. Time = 0 h is 4 h after the beginning of the light period, when cultures received either no supplements (control) or NH_3_, dihydrouracil (DHU) or uracil (Ura) at 5 mm final concentration. Expression values for each transcript at 2, 6 or 21 h after supplement addition are relative to those in control plants, normalized on the basis of *ACT2*expression, and are indicated below each band.

The catabolic pathway intermediate DHU induces the expression of the *pydBC* operon encoding PYD2 and PYD3 orthologues in *Brevibacillus agri* (Kao & Hsu, 2003), and DHU, but not Ura, induces *PYD2* and *PYD3* genes, and β-ureidopropionate also induces *PYD3* expression in *S. kluyveri* (Gojkovic *et al*., 2000, 2001). By contrast, the NH_3_ end-product of the catabolic pathway (see Fig. 1) decreases *PYD2* and *PYD3* expression via nitrogen catabolite repression in *S. kluyveri* (Gojkovic *et al*., 2001). In plants, potential regulation of *PYD* genes by pathway intermediates or products has not been investigated previously. The kinetics of induction or repression of *PYD1–3* gene expression in response to feeding of Ura, DHU and NH_3_ in 17-d-old liquid-cultured seedlings are shown in Fig. 4. NH_3_ strongly represses the expression of*PYD1–3* genes two- to threefold 2 h after supplementation. After 6 h, *PYD1* transcripts return to control levels, whereas twofold*PYD2* and *PYD3* repression is still evident. After 21 h, *PYD1–3* transcript levels are about the same as in untreated controls. Ura feeding weakly increased *PYD1*and *PYD3* expression by *c*. 1.5- to twofold by 6 h, after which transcript levels decreased two- to threefold by 21 h, perhaps as a result of the accumulation of pathway intermediates or the NH_3_ end-product. After 6 h, DHU had little effect on *PYD2* expression, but increased *PYD1* and *PYD3* transcript levels *c*. two- and 2.5-fold, respectively. By 21 h, the expression of PYD1 and PYD3 was still *c*. 1.5-fold higher than in control tissues, unlike the observed decrease in the levels of these transcripts in Ura-treated tissues. As DHU is an intermediate in Ura catabolism, it is unclear why the induction of *PYD1* and *PYD3* was stronger and sustained longer with DHU than with Ura. Possible differences in uptake and metabolism of the two nucleobases were not investigated. Schmidt *et al*. (2004) have demonstrated that the main Ura transporters in Arabidopsis, AtUPS1 and AtUPS2, do not transport DHU and Ura differently. Tintemann *et al*. (1985) reported that unlabelled DHU did not affect the uptake of ^14^C-Ura, but markedly reduced the catabolism of labelled Ura in tomato cells, consistent with DHU being an intermediate in the pathway for pyrimidine catabolism in plants. Tintemann *et al*. (1985) also demonstrated that no labelling of Ura via the potentially reversible DHPDH step was observed with labelled DHU. Thus, exogenous DHU may have resulted in the accumulation of Ura, with altered Ura and DHU pool sizes and *PYD* expression patterns within seedlings in the present experiment. The effects of other pathway intermediates and products (e.g. β-ureidopropionate, β-alanine) on *PYD1–3* expression were not studied here.

The apparent regulation of *PYD* expression by pathway intermediates (Ura and DHU induction; NH_3_ repression) has not been reported previously, and may represent a major mechanism for the control of catabolic pathway activities in plants. Evidence for direct metabolic control of catabolic pathway enzymes is lacking. The heterologously expressed β-UPase (PYD3; Walsh *et al*., 2001) was found to be insensitive, even to 2 mm concentrations of substrates or substrate analogues, such as β-ureidopropionate, β-ureidoisobutyrate and *N*-carbamoyl aspartate, and to the reaction products β-alanine and β-AIB. Plant DHPDH and DHPase have not been purified or biochemically characterized to date.

Metabolic regulation of *PYD1–3* expression by catabolic pathway intermediates may explain the observed *PYD* expression profiles seen in plants grown in media containing different amounts of nitrogen or in Ura as the sole nitrogen source in the present study (Fig. 3), as follows. Under moderate nitrogen limitation (0.4 mm nitrogen), removal of NH_3_, resulting from its assimilation into general nitrogen metabolism, may relieve the repression of *PYD* genes before the depletion of nucleotide pools as a nitrogen source. Further degradation of available pyrimidines under extreme nitrogen limitation (treatment without nitrogen) may lower their cellular titres of Ura below levels that normally would induce *PYD* expression, decreasing *PYD* transcript levels. Indeed, in a similar experiment, *PYRB* expression was unchanged in 3 mm nitrogen treatment, 0.4 mm nitrogen treatment and 1 mm Ura plants, but increased *c*. fivefold in the seedlings grown without treatment, suggesting that pyrimidines were limiting for growth in these plants (Chen & Slocum, 2008). Increased expression of *PYD* genes when Ura is provided as the sole treatment source would result from the induction of *PYD* genes by Ura or catabolic pathway intermediates, accompanied by efficient assimilation of NH_3_, preventing repression of *PYD* expression by this end-product.

The induction of *PYD* expression after 6 h of 5 mm Ura supplementation (Fig. 4) also provides a probable explanation for the apparently high [2-^14^C]-Ura degradation activities in seedlings grown without nitrogen (Table 1), in which *PYD* expression is barely detectable (Fig. 4). Catabolic pathway activity was measured over a period of 16 h in tissues immersed in reaction buffer containing 1 µCi of [2-^14^C]-Ura + 1 mm unlabelled Ura. During this time, it seems likely that Ura may have induced the expression of the *PYD* genes, resulting in higher catabolic activities than were present in the tissue when the assay was initiated. A relatively long assay time was required for intact Arabidopsis tissues, in which rates of incorporation and catabolism of labelled Ura were relatively slow compared with those of suspension-cultured cells used in some studies (Lesley *et al*., 1980; Sasamoto *et al*., 1987), although considerably shorter than the incubation time of 48 h used in a previous study with intact seedlings (Evans & Axelrod, 1961).

### Localization of PYD1- PYD2- and PYD3-GFP fusion proteins in plant cells

**Fig. 5:**
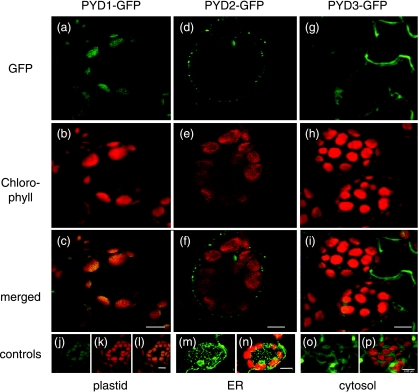
Localization of PYD1-GFP, PYD2-GFP and PYD3-GFP in mesophyll cells of stably transformed Arabidopsis. (a) PYD1-GFP, GFP fluorescence signal (green); (b) PYD1-GFP, chlorophyll autofluorescence signal (red); (c) PYD1-GFP, overlay of GFP and chlorophyll fluorescence signals; (d) PYD2-GFP, GFP fluorescence signal; (e) PYD2-GFP, chlorophyll autofluorescence signal; (f) PYD2-GFP, overlay of GFP and chlorophyll fluorescence signals; (g) PYD3-GFP, GFP fluorescence signal; (h) PYD3-GFP, chlorophyll autofluorescence signal; (i) PYD3-GFP, overlay of GFP and chlorophyll fluorescence signals. (j–l) AMK2-GFP, plastid control: GFP fluorescence signal (green), chlorophyll autofluorescence signal (red) and overlay (Lange *et al*., 2008); (m, n) *mgfp4*-ER, endoplasmic reticulum (ER) control: GFP fluorescence signal (green) and overlay with chlorophyll autofluorescence signal (red) (Haseloff 1998); (o, p) GFP only, cytosolic control: GFP fluorescence signal (green) and overlay with chlorophyll autofluorescence signal (red); Bar, 8 µm.

Lack of detectable DHPDH activity has precluded the localization of this enzyme in subcellular fractions of plant cells, but DHPase and β-UPase activities have been reported to be cytosolic or nuclear in tomato cells (Tintemann *et al*., 1987). Targeting prediction programmes, based on deduced amino acid sequences for these enzymes, predict a plastid localization for DHPDH, the localization of DHPase in the secretory system and a cytosolic localization for β-UPase. For conclusive localization of these enzymes *in vivo*, we cloned C-terminal GFP-fusion constructs with full-length coding sequences of *PYD1*, *PYD2* and*PYD3* genes and stably expressed them in Arabidopsis. Confocal microscopy revealed a plastid localization of the PYD1-GFP fusion (Fig. 5a–c), in agreement with the predicted plastid localization of this enzyme. Localization of PYD2-GFP within the secretory system (Fig. 5d–f) and PYD3-GFP in the cytosol (Fig. 5 g–i) also agrees with targeting predictions. The distribution of these enzymes within several different subcellular compartments suggests a potentially complex regulation. As nucleobases and other pathway intermediates and products have been shown to regulate *PYD* expression, transporters which facilitate the movements of these molecules between these subcellular compartments may also play an important role in regulating pyrimidine degradation in plants.

### Isolation and characterization of *pyd* mutants

As PYD1, PYD2 and PYD3 are encoded by single genes in Arabidopsis, the catabolic pathway is amenable to genetic manipulation. Plants with insertions in *PYD1*, *PYD2* and *PYD3*were screened for homozygosity for the respective T-DNA insertion, as described in Materials and Methods. In order to determine whether T-DNA insertions reduced respective *PYD* transcript levels, quantitative real-time RT-PCR analyses were performed with 16-d-old seedlings grown aseptically on plates containing 1/2 × MS medium and 0.5% sucrose. As shown in Table 2, *PYD1*, *PYD2* and *PYD3* transcripts were easily detected in wild-type seedlings, but no significant expression of these genes was measured in the respective *pyd1-1*,*pyd1-2*,*pyd2-1*,*pyd2-2*and *pyd3* mutants. Thus, they can be regarded as knockouts for their respective gene products.

**Table 2:** Quantitative real-time reverse transcriptase-polymerase chain reaction (RT-PCR) analysis of *PYD* expression in wild-type and *pyd* mutants

	Relative expression (*E*^−Δ*C*t^)		
Sample	*PYD1*	*PYD2*	*PYD3*
Wild-type	0.150 ± 0.0084	0.070 ± 0.0044	0.037 ± 0.0022
*pyd1-1*	0.003 ± 0.0004**	0.079 ± 0.0146	0.044 ± 0.0070
*pyd1-2*	0.003 ± 0.0012**	0.076 ± 0.0047	0.057 ± 0.0153
*pyd2-1*	0.103 ± 0.0084*	0.008 ± 0.0040**	0.023 ± 0.0013*
*pyd2-2*	0.103 ± 0.0195*	0.000 ± 0.0000**	0.023 ± 0.0031*
*pyd3*	0.080 ± 0.0039**	0.040 ± 0.0010**	0.001 ± 0.0001**

Values are expressed as the difference in the *C*t value relative to that of elongation factor 1α*(EF1α)* taken to the power of efficiency. Each value represents the mean ± standard error for six individual seedlings. Measurements were repeated twice.

* Significant differences (*P* < 0.05) using unpaired two-tailed *t*-tests.

** Highly significant differences (*P* < 0.001).

To analyse whether the absence of individual *PYD1*, *PYD2*or *PYD3* expression results in altered expression of other *PYD* genes, transcript levels for all *PYD* genes were analysed in each of the *pyd* mutant lines. When the expression of *PYD1*is absent, the expression of other*PYD* genes clearly remains unaffected (Table 2); however, disrupted expression of either *PYD2*or *PYD3*results in reduced expression of the other two *PYD* genes by 31–47% in both mutants (Table 2). As described above, feeding of wild-type plants with Ura or DHU intermediates and the NH_3_ end-product of the catabolic pathway alters *PYD* expression, although the precise mechanisms by which this occurs are unknown. The availability of Arabidopsi*s pyd*mutants will facilitate analyses of the metabolic regulation of *PYD* expression in the future.

**Fig. 6:**
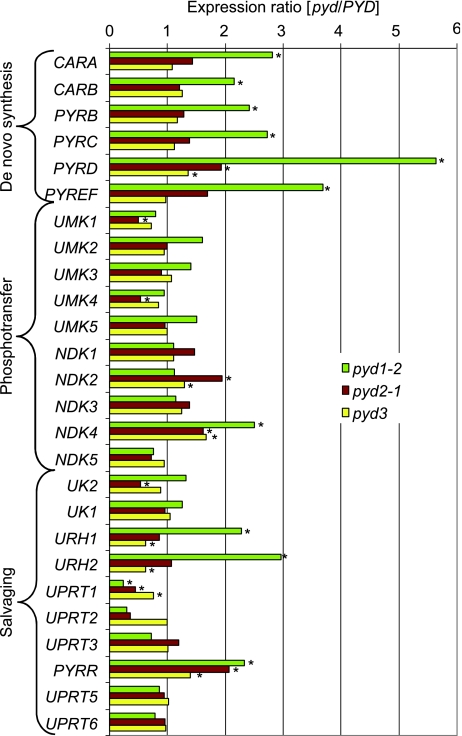
Relative transcript levels of pyrimidine nucleotide metabolism genes in *pyd* mutants. Arabidopsis seedlings (16-d-old) grown on plates containing half-strength Murashige and Skoog (MS) medium with 0.5% sucrose were analysed by quantitative real-time reverse transcriptase-polymerase chain reaction (RT-PCR). Values are calculated as the difference of the *C*t value from *EF1α* taken to the power of efficiency. Each bar represents the expression of the gene in question in the respective mutant relative to the expression in the wild-type. Each bar represents the mean values of six individual seedlings. Measurements were repeated twice. Significant differences (*P* < 0.05) using unpaired two-tailed *t-*tests are marked with an asterisk. *CARA*, carbamoylphosphate synthase, small subunit EC 6.3.5.5; *CARB*, carbamoylphosphate synthase, large subunit EC 6.3.5.5; *NDK*, nucleoside diphosphate kinase EC 2.7.4.6; *PYRB*, aspartate transcarbamoylase EC 2.1.3.2; *PYRC*, dihydroorotase EC 3.5.2.3; *PYRD*, dihydroorotate dehydrogenase EC 1.3.99.11; *PYRFF*, UMP synthase EC 2.4.2.10 and EC 4.1.1.23; *PYRR*, uracil phosphoribosyl transferase EC 2.4.2.9; *UK*, uridine kinase EC 2.7.1.48; *UMK*, uridine monophosphate kinase EC 2.7.4.4; *UPRT*, uracil phosphoribosyl transferase-like EC 2.4.2.9; *URH*, uridine nucleosidase EC 3.2.2.3.

To analyse whether other genes involved in pyrimidine nucleotide metabolism are affected by altered *PYD* gene expression, the expression of genes encoding enzymes involved in *de novo* synthesis, phosphotransfer reactions and pyrimidine salvaging was measured. Expression of all genes could be detected in 16-d-old wild-type plants grown under normal conditions on 1/2 × MS medium containing 0.5% sucrose. As none of the *pyd*mutants exhibit a phenotype, it is not surprising that differences in expression levels for most tested transcripts are relatively minor, compared with wild-type plants (Fig. 6). Nevertheless, markedly increased expression (two- to fivefold) of all *de novo* pathway genes is seen in *pyd1* mutants. Up-regulation of these genes in *pyd1* mutants seems counter-intuitive: potentially increased *de novo* synthesis in tissues with limited pyrimidine catabolism. Other significant expression data for *pyd1* include: a two- to threefold increase in *URH1* and *URH2*, encoding uridine nucleosidases, which metabolize uridine to Ura; a twofold, coordinately down-regulated expression of putative dual-domain uridine kinase/Ura phosphoribosyltransferases *UPRT1* and *UPRT2* (Islam *et al*., 2007), which catalyse the formation of UMP from Ura and uridine; a more than twofold up-regulation of *PYRR*, which would be expected to increase UPRTase activities and salvaging of Ura to UMP. It has been reported from various plant species that uridine is more efficiently salvaged than Ura. This is attributed, in part, to markedly higher uridine-cytidine kinase activities, compared with those of UPRTase, and to low or undetectable uridine nucleosidase activities (reviewed in Stasolla *et al*., 2003). The altered salvage pathway gene expression profiles in the *pyd1* mutants are consistent with a decreased ability to salvage uridine to UMP and increased conversion of uridine to Ura. This difference may explain the observed accumulation of Ura in these plants, compared with *pyd2* and *pyd3* mutants (see below). The reasons for these dynamic changes in synthesis and salvage pathway gene expression in *pyd1* mutants, with reduced catabolic pathway activities, but not in *pyd2* and *pyd3* lines, in which catabolic activities are essentially absent (see below), are unclear and will require further investigation.

### Ura catabolism is impaired in *pyd* mutants

**Fig. 7:**
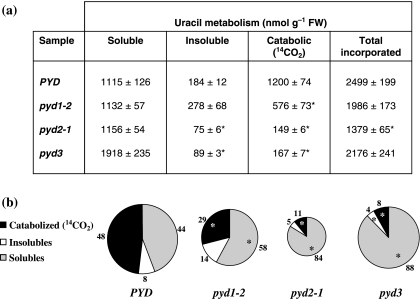
[2-^14^C]-Uracil (Ura) metabolism by *Arabidopsis* seedlings. (a) Total incorporated label and distribution within catabolic (^14^CO_2_), perchloric acid (PCA)-soluble (nucleobases, nucleosides, nucleotides, nucleoside diphosphate sugars) and PCA-insoluble (DNA and RNA) pools. Data are expressed on a nmol Ura g^−1^ fresh weight basis and represent the means ± standard error of three biological replicates. Unpaired two-tailed *t*-tests were used to compare labelling data for wild-type *PYD* and *pyd* mutant plants. Significant differences with *P* < 0.05 are labelled with an asterisk. (b) Metabolite labelling as a percentage of total incorporated Ura. The size of the circle represents the amount of Ura that was taken up by the respective seedling culture.

**Fig. 8:**
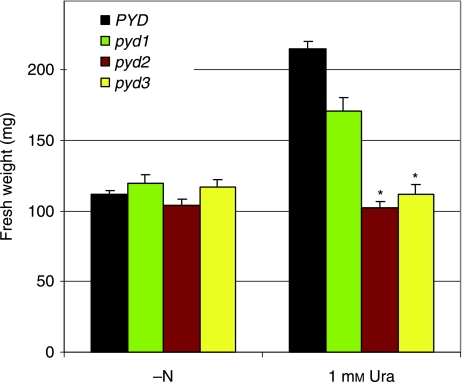
Growth of wild-type *PYD* and *pyd* mutant Arabidopsis plants in sterile culture on media containing either no nitrogen (−N), or 1 mm uracil (1 mm Ura). Complete rosettes were harvested after 38 d of growth and the fresh weight was recorded. Values represent the means ± standard error of at least 22 individual seedlings. Significant differences compared with *PYD* in each group (*P* < 0.05) using unpaired two-tailed *t-*tests are marked with an asterisk.

In order to determine whether the absence of *PYD1*, *PYD2* or *PYD3* expression results in altered Ura metabolism, we measured pyrimidine levels of *pyd* mutants grown for 9 d in liquid culture. Although pyrimidine nucleotide and uridine levels are unchanged in all *pyd* mutants (data not shown), there is a strong and significant accumulation of Ura, with the largest increase occurring in *pyd1*. Although control plants contain 16 ± 7 µmol Ura g^−1^ FW, *pyd1* mutants accumulate 126 ± 19, *pyd2* mutants 41 ± 9 and *pyd3* mutants 67 ± 8 µmol Ura g^−1^ FW, respectively. We further analysed radiolabelled metabolite profiles after feeding of [2-^14^C]-Ura to liquid-cultured seedlings. Figure 7shows that, in wild-type (*PYD*) plants, 48% of incorporated [2-^14^C]-Ura is catabolized to ^14^CO_2_. In *pyd2* and *pyd3* mutants, Ura degradation is nearly undetectable (11% and 8% of incorporated label, respectively), and possible mechanisms by which a small amount of Ura is apparently catabolized to ^14^CO_2_ in these mutants are unknown. The absence of *PYD2* and *PYD3* transcripts in these plants (Table 2) and their inability to grow on Ura as a sole nitrogen source (Fig. 8) clearly confirm that Ura degradation in plants is dependent on the activities of DHPase (PYD2) and β-UPase (PYD3). This also demonstrates that the Arabidopsis homologue to the recently described, completely different and unrelated *PYD3* gene encoding β-alanine synthase from *S. kluyveri* (Andersen *et al*., 2008a) is not able to substitute the missing β-UPase expression in the *pyd3* mutants.

By contrast, 29% of incorporated [2-^14^C]-Ura was released as ^14^CO_2_ by the *pyd1* mutant, representing only a 40% decrease in catabolic pathway activity compared with wild-type plants. The strongly reduced catabolism of Ura in the *pyd1* mutant unequivocally demonstrates the involvement of PYD1 in pyrimidine degradation. However, significant production of ^14^CO_2_ in the *pyd1* mutant suggests an incomplete loss of PYD1 function, or the possibility of additional, unknown mechanisms for Ura catabolism in Arabidopsis.

In comparison with wild-type plants, none of the mutants exhibited obvious growth or developmental differences under normal growing conditions, in either sterile culture or on soil (data not shown). In order to further confirm the importance of *PYD1*, *PYD2* and *PYD3* expression for Ura degradation and the role of the catabolic pathway in the recycling of nitrogen from nucleobases to general nitrogen metabolism, wild-type and *pyd* mutant seedlings were grown on media containing either no nitrogen or 1 mm Ura as a sole nitrogen source. As shown in Fig. 8, growth of all plants on medium without nitrogen was not significantly different, and limited seedling growth can be explained by the salvaging of seed nitrogen reserves and the small amount of nitrogen in the Murashige and Skoog (1962) vitamin solution (*c*. 30 µm). Growth of *pyd2* and *pyd3* plants in medium containing Ura as the sole nitrogen source was not significantly different from that in the medium without nitrogen, unequivocally demonstrating that, without PYD2 or PYD3 function, Ura degradation is not possible in plants. Interestingly, *pyd1* mutants were able to grow on 1 mm Ura medium, clearly supporting limited Ura catabolism in these plants (Table 3).

**Table 3:** Compilation of the genes and mutants of the reductive pathway of pyrimidine degradation in *Arabidopsis thaliana*, including a summary of the mutant phenotypes

Enzyme	Abbreviation	Gene	EC number	Accession	Mutant	Uracil accumulation	CO_2_ release (% control)	Growth on uracil as sole nitrogen source
Dihydropyrimidine dehydrogenase	DHPDH	*PYD1*	EC 1.3.1.2	At3g17810	SALK_083897 (*pyd1-2*)	+	60%	+
					GABI_251F09 (*pyd1-1*)	+	nd	+
Dihydropyrimidinase	DHPase	*PYD2*	EC 3.5.2.2	At5g12200	SALK_038919 (*pyd2-1*)	+	11%	–
					GABI_114F11 (*pyd2-2*)	+	nd	–
β-ureidopropionase	β-UPase	*PYD3*	EC 3.5.1.6	At5g64370	SALK_016594 (*pyd3*)	+	9%	–

nd, not determined.

Although T-DNA insertions within genes frequently result in a complete loss of function associated with the protein product, our initial determination that *PYD1* expression was absent from *pyd1* mutants (Table 2) was based on the use of PCR primers that amplified sequences downstream of the T-DNA insertion site (Table S2). If *PYD1* transcripts terminating at the insertion site produced truncated, functional PYD1 proteins with reduced DHPDH activity, this might explain the apparent Ura catabolism in these mutants. In plants that had been grown on soil for 4 wk, we used quantitative real-time RT-PCR to analyse the possible existence of truncated*PYD1*transcripts in the *pyd1* mutants, using three primer pairs to detect transcripts upstream of the T-DNA insertions and a primer pair downstream of the insertion (Table S4, see Supporting Information). As shown in Table 4, the *PYD1* transcript was not present when the primer pair downstream of the insertions was used for amplification. Amplification using primer pairs upstream of the insertions indicated that the expression of truncated transcripts did occur, at levels representing *c*. 50% of those seen in wild-type plants. In the *pyd1-1* mutant, the T-DNA insertion is in intron 3, whereas, in *pyd1-2*, the insertion is at the beginning of exon 4.

**Table 4:** Quantitative real-time reverse transcriptase-polymerase chain reaction (RT-PCR) analysis of *PYD1* expression in congeneric wild-type and *pyd1* mutants

	Relative expression (*E*^−Δ*C*t^)	
Primer pair	*PYD1*	*pyd1*
PYD1 268–342	0.0102 ± 0.0010	0.0067 ± 0.0012
PYD1 530–590	0.0097 ± 0.0003	0.0057 ± 0.0001
PYD1 613–683	0.0110 ± 0.0007	0.0063 ± 0.0002
PYD1 1054–1143	0.0265 ± 0.0011	0.0003 ± 0.0002

Values are expressed as the difference in the *C*t value relative to that of *EF1α*taken to the power of efficiency. Each value represents the mean ± standard error for three individual seedlings. Measurements were repeated twice.

Analysis of the *PYD1* gene indicates that, in either *pyd1* line, transcripts terminating at the T-DNA insertion site could potentially include coding regions of exons 1–3, encoding PYD1 proteins truncated at or before residue E239. Searches for motifs (InterPro; The UniProt Consortium, 2007) and conserved domains (CDD; Marchler-Bauer *et al*., 2007) revealed that such truncated PYD1 would not include the complete DHPDH Ura/flavin mononucleotide-binding domain, including active site residues N256 and T257, or two highly conserved subunit interaction domains (T280–I299; E328–H349). Thus, it is very unlikely that the truncated PYD1 proteins would be functional, based on structural studies with animal DHPDH (Schnackerz *et al*., 2004; Protein Data Bank ID: 1GTE), although DHPDH activities for such proteins cannot be precluded at this time. As PYD1 antibodies are presently unavailable, it was not possible to determine whether truncated PYD1 proteins were even made in the *pyd1* mutants. Previous investigators have been unable to measure DHPDH in plant extracts (for example, Tintemann *et al*., 1985), or for heterologously expressed Arabidopsis PYD1 protein (Z. Gojkovic, ZGene, Horsholm, Denmark, pers. comm.) or *Brevibacillus agri* PydA (Kao & Hsu, 2003). This may be explained by the absence of an N-terminal (GltD) domain containing FeS clusters and NAD(P)H and flavin adenine dinucleotide cofactor binding sites in the plant PYD1 and bacterial PydA sequences. As this compulsory interaction partner required for the activity of plant PYD1 has not yet been identified, it is not possible to elucidate putative alternative partners in the *pyd1* mutant background.

We further investigated the role of PYD1 in plant pyrimidine catabolism using RNAi-mediated silencing of *PYD1* expression. The RNAi lines would be expected to produce markedly decreased levels of a full-length, presumably fully functional PYD1 protein. In strongly silenced lines, *PYD1* transcript levels were reduced by 85%, but Ura catabolism was decreased by only 40%, compared with wild-type plants (Fig. S2, Table S5, see Supporting Information). Again, these observations support a role for PYD1 in Ura catabolism but, as in studies with *pyd1* mutants, the absence of assays for DHPDH activity, labelling kinetic data, etc. do not permit us to differentiate between DHPDH-mediated Ura reduction and Ura reduction via additional, unknown activities.

If such alternative, non-PYD1-mediated Ura reduction activities exist in plants, they would require the formation of DHU and β-ureidopropionate intermediates in Ura catabolism, based on previous metabolic labelling studies (Evans & Axelrod, 1961; Tintemann *et al*., 1985), and the observation that *pyd2* and *pyd3* mutants cannot grow on Ura as a nitrogen source, whereas *pyd1* mutants can. The only closely related sequence to *PYD1* in the Arabidopsis genome is dihydroorotate dehydrogenase, the enzyme catalysing the fourth step in the pyrimidine *de novo* synthesis (Zrenner *et al*., 2006). It has been shown that this plant enzyme is located at the outer side of the inner mitochondrial membrane, and there is no further evidence that it might also be involved in pyrimidine degradation (Ullrich *et al*., 2002).

An interesting parallel is seen in the yeast *S. kluyveri*, which has functional PYD2 and PYD3 and can utilize DHU as a nitrogen source (Gojkovic *et al*., 2000, 2001). In contrast with the situation in plants, however, PYD1 orthologues have not been identified in this yeast, and Ura catabolism occurs via a newly described pathway that does not produce DHU as an intermediate (Andersen *et al*., 2008b). Apparently, the truncated PYD2/PYD3 reductive pathway is an evolutionary relic that no longer functions in normal Ura catabolism in this organism. Other alternative pathways for pyrimidine catabolism have been described in bacteria (Loh *et al*., 2006). Exhaustive searches of plant genomes have not revealed plant orthologues of genes and enzymes in these bacterial or yeast pathways (Table S6, see Supporting Information). Further biochemical and metabolic labelling studies, and the development of assays for DHPDH activity and enzyme protein expression and the functional characterization of heterologously expressed DHPDH, are required to more fully understand the role of this enzyme in Ura catabolism in plants.

### Conclusion

During growth and development, the expression of *PYD* genes is coordinated with the expression of genes encoding *de novo* and salvage synthesis pathway enzymes, presumably to maintain pyrimidine homeostasis in response to changing metabolic demands. The catabolic pathway does not appear to be essential under normal growing conditions, as *pyd* mutants exhibit no obvious phenotype. Under conditions of nitrogen limitation, the pathway appears to play a major role in the recycling of pyrimidine nitrogen to general nitrogen metabolism. Metabolic regulation of catabolic pathway genes, including the induction of *PYD* expression by Ura and DHU, and repression by the NH_3_ end-product, is similar to that described in other organisms. Functional analyses of pyrimidine catabolism in Arabidopsis have demonstrated that PYD2 and PYD3 and, to a lesser extent, PYD1 play essential roles in Ura catabolism in this plant, although an unknown, alternative route for Ura catabolism may also be present. Localization of catabolic pathway enzymes in different subcellular compartments suggests a potentially complex regulation of genes and enzymes by pathway intermediates and end-products, about which little is currently known.

## Abbreviations

β-AIB: β-aminoisobutyrate
BAT: β-alanine aminotransferase
CoA: coenzyme A
DHPase: dihydropyrimidinase
DHPDH: dihydropyrimidine dehydrogenase
DHU: dihydrouracil
EF1a: elongation factor 1a
EGTA: ethylene glycol tetraacetic acid
ER: endoplasmic reticulum
GFP: green fluorescent protein
His: histidine
HPLC: high-performance liquid chromatography
MES: 2-[*N*-morpholino]ethanesulphonic acid
MS medium: Murashige and Skoog medium
NCBI: National Center for Biotechnology Information
ORF: open reading frame
PCA: perchloric acid
RNAi: RNA interference
RT-PCR: reverse transcriptase-polymerase chain reaction
TAIR: The *Arabidopsis* Information Resource
Thy: thymine
UDP: uridine diphosphate
UMP: uridine-5′-monophosphate
β-UPase: β-ureidopropionase
UPRTase: uracil phosphoribosyl transferase
Ura: uracil

## Supporting Information

**Table S1** PCR primers used in reverse transcriptase-polymerase chain reaction (RT-PCR) analyses of relative transcript levels for genes encoding enzymes of pyrimidine nucleotide metabolism and nucleobase transporters

**Table S2** PCR primers used in quantitative real-time reverse transcriptase-polymerase chain reaction (RT-PCR) analyses of relative transcript levels for genes of pyrimidine nucleotide metabolism

**Table S3** PCR primers used for genotyping of *pyd* mutants

**Table S4** PCR primers used in quantitative real-time reverse transcriptase-polymerase chain reaction (RT-PCR) analyses of 5′- and 3′-relative transcript levels for the *PYD1* gene and the reference gene *EF1α*

**Table S5** [2-^14^C]-Uracil metabolism in leaves of 9-d-old liquid-cultured seedlings grown in 1/2 × Murashige and Skoog (MS) medium

**Table S6** Compilation of genes and enzymes involved in the different pathways of pyrimidine degradation in various organisms and putative homologues identified in the Arabidopsis genome

**Fig. S1** Phenotype of 6-wk-old Arabidopsis seedlings grown on washed vermiculite potting medium and fertilized with 1/2 × Murashige and Skoog (MS) medium containing 3 mm nitrogen, 0.4 mm nitrogen, no nitrogen (−N) or 1 mm uracil as the sole nitrogen source.

**Fig. S2** Reverse transcriptase-polymerase chain reaction (RT-PCR) analysis of *PYD1* expression in wild-type (Col-0) plants and in two transgenic lines in which RNAi-mediated silencing resulted in an 80−85% reduction in *PYD1* transcript levels, normalized on the basis of the expression of the invariant *ACT2* control.
