# Supplementary material for: A functional analysis of the pyrimidine catabolic pathway in Arabidopsis
Source: New Phytol. 2009 Jul;183(1):117–32. doi: 10.1111/j.1469-8137.2009.02843.x (PMC2713857; doi:10.1111/j.1469-8137.2009.02843.x)
Supplement: Supplementary file 1 [file nph0183-0117-SD1.pdf]

AGI Locus	Gene Name	Forward 5' -> 3'	Reverse 5' -> 3'
At3g18780	*ACT2*	TCCTCACTTTCATCAGCCG	ATTGGTTGAATATCATCAGCC
At3g20330	*PYRB*	GAAGTTGTGACTCAGTATCTCC	CAACTAAGGCTACACTGATGCC
At3g53900	*PYRR*	AACCCTAATCCTTCCTCTTTCC	TAGCAGGTAAAACTGAGGATGC
At3g17810	*PYD1*	TTTCGCCTTGAATCGCTTCTCTGG	AGATACGGTTTTGGCGATGACGGC
At5g12200	*PYD2*	TGGTATTATTGTCGCTGTGC	GGAGTAGGTCATCAGTTACC
At5g64370	*PYD3*	AGAGCCTGTTGATGGCGAATCG	AGAGCCTGTTGATGGCGAATCG
At3g08860	*PYD4*	TAACGGCGGTTAATTCACTA	GGTGTGTTGTAGAAGTGAAAAAGAG
At1g70330	*AtENT1*	CGCTTACATCATCTACTTCACC	AAAACAGGAACCACAAGCAAGGCG
At2g03590	*AtUPS1*	GCACAATAATCGGATTGGTG	ATGTTAAGTATCAGAGCAACTACAAATGC
At2g03530	*AtUPS2*	GTATCGTGCTTAGCCTCG	TGTTCCTATTTCGGTAGATGGACC

Table S1 PCR primers used for semiquantitative RT-PCR analyses of relative transcript levels for genes encoding enzymes of pyrimidine nucleotide metabolism, nucleobase transporters, and the reference gene *ACT2*.
