# Supplementary material for: A functional analysis of the pyrimidine catabolic pathway in Arabidopsis
Source: New Phytol. 2009 Jul;183(1):117–32. doi: 10.1111/j.1469-8137.2009.02843.x (PMC2713857; doi:10.1111/j.1469-8137.2009.02843.x)
Supplement: Supplementary file 2 [file nph0183-0117-SD2.pdf]

**AGI Locus**	**Gene Name**	**Forward 5´->3´**	**Reverse 5´->3´**
At3g27740	*CARA*	GCTCAGAATCACAACTATGCGGT	TGTGTTACTTCTACGCCTCCGG
At1g29900	*CARB*	GAGAAAATCGCGGTGTCCTTC	GTTCCCGAGGTGGCAACTATT
At3g20330	*PYRB*	GGATGAAATCACCGCAGATGTT	GTCTGAAGTAGGCAGCTCTTGGA
At4g22930	*PYRC*	CACATCACAACCATGGATGCTG	ACCCACAGACCCTTCTTTGCA
At5g23300	*PYRD*	GCTTGAAGATATTGCAGCGGTG	TATGATCAGCCCATCCAAGTGC
At3g54470	*PAREF*	ATACTGCCTGATTTCACCCCTG	TGCAATCGCACGGAGCTTA
At3g60180	*UMK1*	TGCTAGAATCGAGCCTGCTTTT	TGGTTCCTGCTCATAATGCGT
At4g25280	*UMK2*	AACCGTGTTGCATTTGAGCG	CGGCCCTGATTACGATTCAAG
At3g18680	*UMK3*	GGATGCTGGCAACTGTGATGA	GGATGCTGGCAACTGTGATGA
At3g10030	*UMK4*	TGGTAATATTTGGTGGCATCGG	CCGCATTGATGTCTATAGCTCGA
At5g26667	*UMK5*	CCGAGCAGCATTTGAAAAAGTT	AACAGGCGCTTCTCCATCTCT
At4g09320	*NDK1*	ATTATGATCAAGCCTGACGGC	GCAGATGACTTCACCGATGAGT
At5g63310	*NDK2*	ACCGGAAGGAACATTGTGCA	TCACGCTTGCCGTTTTCAG
At4g11010	*NDK3*	CCTGTTATTGCCATGGTCTGG	GCTCCAATCAGTTTACGTCCGT
At4g23900	*NDK4*	TGCTTCTAGAGCAGCCAGGTCT	CGGCCTTCGGAGAAGAAA
At1g17410	*NDK5*	CCCTCACAGCATCAGAGCATT	AGCCGTGCACACAGTTCTTCT
At1g73980	*UK1*	GCCCTAAAGAAGCCCTCTCAAG	ACGCCGCCGTGAAGATATTT
At1g26190	*UK2*	TGAAGTAATGGCCCTACCAGATG	AGACGCTCGTAAGAGTGCTTCC
At1g05620	*URH1*	AGGTTTGAGGAAGCGAACGAG	ATCAACCGTCACTGCCACTTTT
At2g36310	*URH2*	GCCTCAAGAGATGGAACGGAA	CGTCCAAGCAACTGATATCGGT
At1g55810	*UPRT1*	ATGCACACTCTAATCCGCGACT	ACCAAACGTATCAATCGATCGG
At3g27440	*UPRT2*	CTTGACCCTGTTCTAGCATCAGG	TGATTCTGGAACTCCCTTGCTT
At3g27190	*UPRT3*	ACGGAGACAATGGAATGCAGC	CGCTCGGAGATATCACTTGGAA
At3g53900	*PYRR*	TTGAAGGAACGTGGTTTGTCTG	GCAGCAATCGCACAAATCAC
At4g26510	*UPRT5*	CAGGAAACTCAGCGGTTGAAG	TGATGTTTCCCTCAGGTACGC
At5g40870	*UPRT6*	CGACATATCTGAACGCCATGTC	GCCGAGTTACCTGTGGCTAAGA
At3g17810	*PYD1*	AAAACCCTTTGCGCTGAGCT	AAGTACTGCAGCGAATGCCCT
At5g12200	*PYD2*	AATGGTGTTAATGGCCTTGAGG	CATCGTGTCCCATATCAAGTGC
At5g64370	*PYD3*	ACCCTTCAGCAACTGTTGGTGA	CGGCATTTCGTGCCTCAATA
At5g60390	*EF1a*	TGAGCACGCTCTTCTTGCTTTCA	GGTGGTGGCATCCATCTTGTTACA
		TGGTGACGCTGGTATGGTTA	TCCTTCTTGTCCACGCTCTT
At3g18780	*ACT2*	TCCCTCAGCACATTCCAGCAGAT	AACGATTCCTGGACCTGCCTCATC
		ACCAGCTCTTCCATCGAGAA	GGGCATCTGAATCTCTCAGC
At4g05320	*UBQ10*	CACACTCCACTTGGTCTTGCGT	TGGTCTTTCCGGTGAGAGTCTTCA
		GTCGACCCTTCACTTGGTGT	CCTTGACGTTGTCAATGGTG

Table S2 PCR primers used in quantitative real-time RT-PCR analyses of relative transcript levels for genes of pyrimidine nucleotide metabolism and three reference genes.
