# Supplementary material for: A functional analysis of the pyrimidine catabolic pathway in Arabidopsis
Source: New Phytol. 2009 Jul;183(1):117–32. doi: 10.1111/j.1469-8137.2009.02843.x (PMC2713857; doi:10.1111/j.1469-8137.2009.02843.x)
Supplement: Supplementary file 3 [file nph0183-0117-SD3.pdf]

Insert	Primer Name	Forward 5´->3´	Primer Name	Reverse 5´->3´
pyd1-1	GABI_251F09L	CTCGTGTCCTCATGGTATGC	GABI_251F09R	CCTTATAAGAGTATCCCCCTGGAG
pyd1-2	SALK_083897L	CCTGGTTTTTCAACCATTTTGT	SALK_083897R	CGGATTTTAGTGACACCCTAGC
pyd2-1	SALK_038919L	CTGACTTGACTGGTTTCTTGCTT	SALK_038919R	AAAAAGAAGCTTCGAAAGAGCAT
pyd2-2	GABI_114F11L	CTCTTGCCTCTGTAAAGCTTTGTT	GABI_114F11R	TGGAAAGGGCATCTTGTAGG
pyd3	SALK_016594L	TATTACTTGCTTGGTGGCAGAA	SALK_016594R	GCCTGCAAGTTTTAAGGAACAT
GABI	GABI_L	CCCATTTGGACGTGAATGTAGACAC		
SALK	SALK_Lbb1	GCGTGGACCGCTTGCTGCAACT		

Table S3 PCR primers used for genotyping of *pyd* mutants.
