# Supplementary material for: A functional analysis of the pyrimidine catabolic pathway in Arabidopsis
Source: New Phytol. 2009 Jul;183(1):117–32. doi: 10.1111/j.1469-8137.2009.02843.x (PMC2713857; doi:10.1111/j.1469-8137.2009.02843.x)
Supplement: Supplementary file 4 [file nph0183-0117-SD4.pdf]

Primer Name	Forward 5´->3´	Primer Name	Reverse 5´->3´
PYD1-268F	GTCATCGCCAAAACCGTATC	PYD1-342R	GGTTCGTAGCCGAGCATATC
PYD1-530F	TTGAGCAAACTGGTGTTGATGC	PYD1-590R	TCTGGCATACCATGAGGACA
PYD1-613F	GGACAAGATTGTGCGCTTCT	PYD1-683R	GCCCATACAGGAACTGTAGCTT
PYD1-1054F	AAAACCCTTTGCGCTGAGCT	PYD1-1142R	AAGTACTGCAGCGAATGCCCT
EF1α-471F	TGAGCACGCTCTTCTTGCTTTCA	EF1α-546R	GGTGGTGGCATCCATCTTGTTACA
EF1α-1221F	TGGTGACGCTGGTATGGTTA	EF1α-1367	TCCTTCTTGTCCACGCTCTT

Table S4 PCR primers used in quantitative real-time RT-PCR analyses of 5´- and 3´-relative transcript levels for the *PYD1* gene and the reference gene *EF1α*.
