# Supplementary material for: A functional analysis of the pyrimidine catabolic pathway in Arabidopsis
Source: New Phytol. 2009 Jul;183(1):117–32. doi: 10.1111/j.1469-8137.2009.02843.x (PMC2713857; doi:10.1111/j.1469-8137.2009.02843.x)
Supplement: Supplementary file 5 [file nph0183-0117-SD5.pdf]

Sample	Soluble	Insoluble	Catabolic ($^{14}CO_2$)	Total Incorporated
WT Shoot	740.9 ± 16.4 [82.1]	113.9 ± 29.7 [12.6]	47.3 ± 6.4 [5.2]	902.1 ± 115.9
PYD1,3-1/3 Shoot	791.4 ± 64.5 [83.9]	122.9 ± 23.9 [13.0]	29.3 ± 4.5 [3.1]	943.6 ± 80.4

Table S5 [2-^{14}C]-Uracil metabolism in leaves of 9-day-old liquid-cultured seedlings grown in 1/2x MS medium. Catabolic pathway activities were estimated by $^{14}CO_2$ production (see Fig. 1) in wild-type Col-0 and RNAi line *PYD1,3-1/3*, which expresses *PYD1* at approximately 15% of levels found in wild type plants. Soluble, perchloric acid-soluble fraction; Insoluble, perchloric acid-insoluble fraction. Values are means ± standard error for four replicates. Significant differences ($P < 0.05$) using unpaired, two-tailed *t*-tests, are marked with an asterisk. Values in brackets indicate the percentage of total incorporated Ura.
