# Supplementary material for: A functional analysis of the pyrimidine catabolic pathway in Arabidopsis
Source: New Phytol. 2009 Jul;183(1):117–32. doi: 10.1111/j.1469-8137.2009.02843.x (PMC2713857; doi:10.1111/j.1469-8137.2009.02843.x)
Supplement: Supplementary file 6 [file nph0183-0117-SD6.pdf]

Enzyme	Gene	Accession	Organism	Reference	Arabidopsis close homologue
**Reductive pathway**					
Dihydropyrimidine dehydrogenase	*DPYD*	NM_214044	*Sus scrofa*	Yokota et al. 1994	At3g17810
Dihydropyrimidinase		At5g12200	*Arabidopsis thaliana*	Gojkovic et al. 2003	At5g12200
β-ureidopropionase		At5g64370	*Arabidopsis thaliana*	Walsh et al. 2001	At5g64370
β-alanine synthase		AAK60518	*Saccharomyces kluyveri*	Andersen B et al. 2008	At5g43600
**Oxidative pathway**					
Uracil dehydrogenase	no gene identified yet				
Barbiturase	*bar*	AJ320520	*Rhodococcus erythropolis*	Soong et al. 2002	none
Ureidomalonase	no gene identified yet				
**RUT pathway**					
Monooxygenase	*rutA*	YP_002555908	*Escherichia coli*	Loh et al. 2006	none
Isochorismatase (putative)	*rutB*	YP_002386513	*Escherichia coli*	Loh et al. 2006	none
Endoribonuclease	*rutC*	YP_002386512	*Escherichia coli*	Loh et al. 2006	none
Hydrolase (putative)	*rutD*	YP_002386511	*Escherichia coli*	Loh et al. 2006	none
Nitroreductase	*rutE*	YP_002386510	*Escherichia coli*	Loh et al. 2006	none
Flavin reductase	*rutF*	YP_002386509	*Escherichia coli*	Loh et al. 2006	none
Pyrimidine permease	*rutG*	YP_002555902	*Escherichia coli*	Loh et al. 2006	none
**URC pathway**					
Cyclohydrolase (putative)	*urc1*	AY154654	*Saccharomyces kluyveri*	Andersen et al. 2008	none
Transcription factor	*urc2*	AY154653	*Saccharomyces kluyveri*	Andersen et al. 2008	none
Urea amidohydrolase, urea carboxylase	*urc3+5*	DQ512718	*Saccharomyces kluyveri*	Andersen et al. 2008	partial homology to various carboxylases
unknown	*urc4*	DQ512719	*Saccharomyces kluyveri*	Andersen et al. 2008	none
Uracil phosphoribosyltransferase (UPRT)	*urc6*	DQ512720	*Saccharomyces kluyveri*	Andersen et al. 2008	6 different proteins with UPRT domain

Table S6 Compilation of genes and enzymes involved in the different pathways of pyrimidine degradation in various organisms and putative homologues identified in the Arabidopsis genome.
