# Supplementary material for: A functional analysis of the pyrimidine catabolic pathway in Arabidopsis
Source: New Phytol. 2009 Jul;183(1):117–32. doi: 10.1111/j.1469-8137.2009.02843.x (PMC2713857; doi:10.1111/j.1469-8137.2009.02843.x)
Supplement: Supplementary file 7 [file nph0183-0117-SD7.pdf]

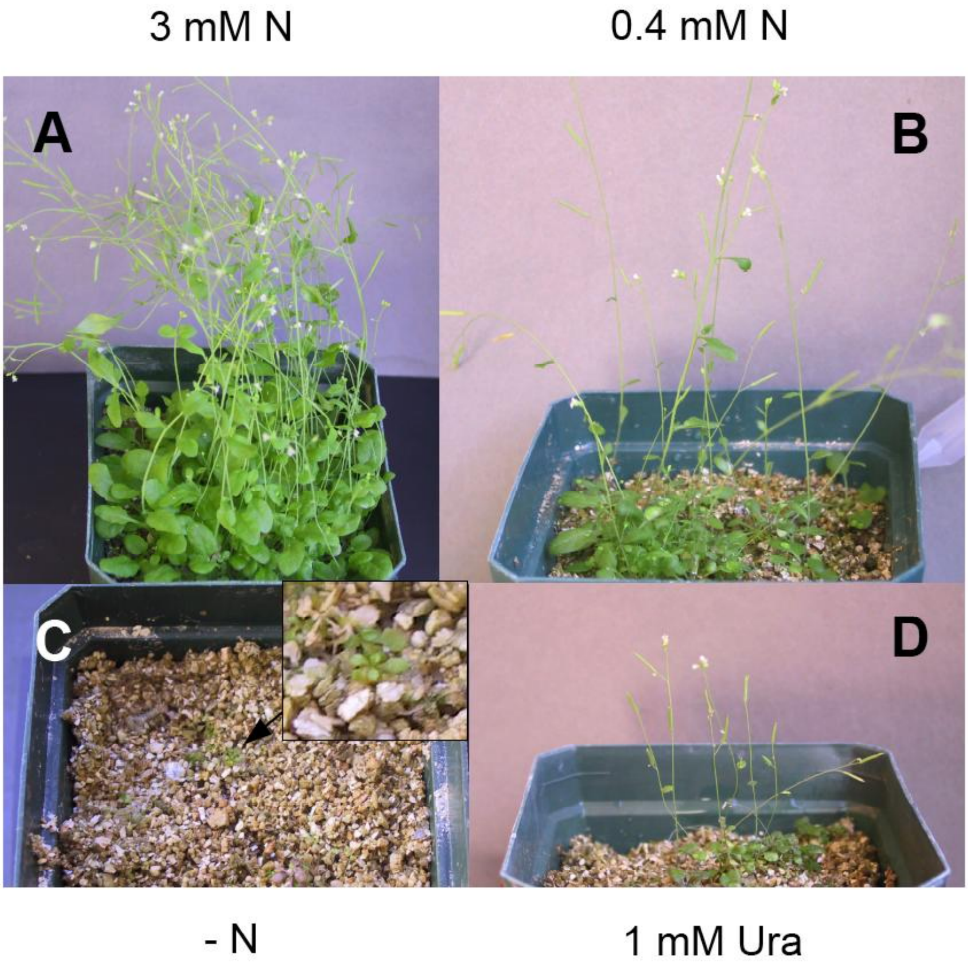

Fig. S1 Phenotype of 6-week-old Arabidopsis seedlings grown on washed vermiculite potting medium and fertilized weekly with equal volumes of 1/2x MS medium containing either 3 mM N (1 mM KNO_3 and 1 mM NH_4NO_3), 0.4 mM N (0.13 mM KNO_3 and 0.13 mM NH_4NO_3), no nitrogen (- N) or 1 mM uracil (1 mM Ura) as the sole N source.
