# Supplementary material for: A functional analysis of the pyrimidine catabolic pathway in Arabidopsis
Source: New Phytol. 2009 Jul;183(1):117–32. doi: 10.1111/j.1469-8137.2009.02843.x (PMC2713857; doi:10.1111/j.1469-8137.2009.02843.x)
Supplement: Supplementary file 8 [file nph0183-0117-SD8.pdf]

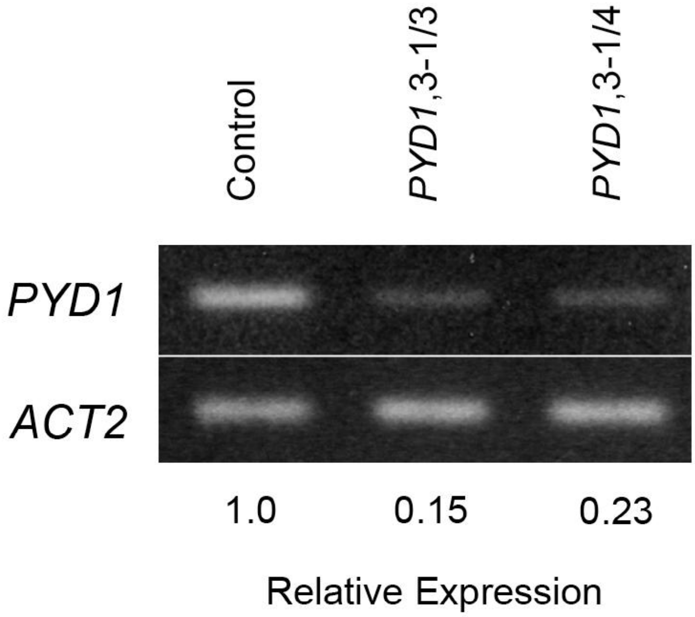

Fig. S2 RT-PCR analysis of *PYD1* expression in wild-type (Col-0) plants and in two transgenic lines in which RNAi-mediated silencing resulted in 80-85% reduction in *PYD1* transcript levels, normalized on the basis expression of the invariant *ACT2* control.
